# A Nervous System-Specific Model of Creatine Transporter Deficiency Recapitulates the Cognitive Endophenotype of the Disease: a Longitudinal Study

**DOI:** 10.1038/s41598-018-37303-1

**Published:** 2019-01-11

**Authors:** Angelo Molinaro, Maria Grazia Alessandrì, Elena Putignano, Vincenzo Leuzzi, Giovanni Cioni, Laura Baroncelli, Tommaso Pizzorusso

**Affiliations:** 1 Institute of Neuroscience, National Research Council (CNR), I-56124 Pisa, Italy; 2 Department of Neuroscience, Psychology, Drug Research and Child Health NEUROFARBA, University of Florence, I-50135 Florence, Italy; 3 Department of Developmental Neuroscience, IRCCS Stella Maris Foundation, I-56128 Pisa, Italy; 4 Department of Paediatrics, Child Neurology and Psychiatry, Sapienza University of Rome, I-00184 Rome, Italy; 5 Department of Clinical and Experimental Medicine, University of Pisa, I-56126 Pisa, Italy

## Abstract

Mutations in creatine (Cr) transporter (CrT) gene lead to cerebral creatine deficiency syndrome-1 (CTD), an orphan neurodevelopmental disorder presenting with brain Cr deficiency, intellectual disability, seizures, movement and autistic-like behavioral disturbances, language and speech impairment. We have recently generated a murine model of CTD obtained by ubiquitous deletion of 5–7 exons in the CrT gene. These mice showed a marked Cr depletion, associated to early and progressive cognitive impairment, and autistic-like defects, thus resembling the key features of human CTD. Given the importance of extraneural dysfunctions in neurodevelopmental disorders, here we analyzed the specific role of neural Cr in the CTD phenotype. We induced the conditional deletion of *Slc6a8* gene in neuronal and glial cells by crossing CrT floxed mice with the Nestin::Cre recombinase Tg (Nes-cre) 1Kln mouse. We report that nervous system-specific Cr depletion leads to a progressive cognitive regression starting in the adult age. No autistic-like features, including repetitive and stereotyped movements, routines and rituals, are present in this model. These results indicate that Cr depletion in the nervous system is a pivotal cause of the CTD pathological phenotype, in particular with regard to the cognitive domain, but extraneural actors also play a role.

## Introduction

Creatine (Cr) transporter (CrT) deficiency syndrome (CTD, OMIM #300352) is an X-linked disease characterized by primary cerebral Cr deficiency. The clinical picture of this metabolic disorder, affecting about 1% of males with non-syndromic mental disability, includes mental retardation, autistic-like traits, language and speech disturbances, seizures and movement disorders^1,2^. In contrast to the other two Cr deficiency syndromes^3–5^, the attempts to rescue Cr content in the CTD brain by nutritional supplementation have been of limited success and no satisfactory treatments are available for CTD disorder^2,6–10^.

Preclinical animal models are crucial tools to dissect disease pathogenic mechanisms and develop new therapeutic strategies. Two germline murine models and three conditional models of CTD are available so far^11–15^. Whole-body knockout mice (CrT^−/y^) exhibited Cr deficiency in the brain and peripheral tissue. Behavioral analysis revealed that CrT^−/y^ mice show early global cognitive deterioration and autistic-like behavior. A progressive decline of cognitive abilities is detectable in these mutant mice, suggesting that age could be a key feature of the disease^11,12,14^. The broad spectrum of phenotypes displayed by CrT^−/y^ mice reproduced the key clinical features of CTD patients and strongly established the face validity and the utility of this model for translational studies.

Since it has been recently reported that extraneural dysfunctions could underlie behavioral deficits in other mouse models of neurodevelopmental disorders^16,17^, we analyzed the phenotype of a nervous system-specific CrT knockout mouse^14,18^ (nes-CrT^−/y^) with the aim to dissect the role of neural Cr in CTD. Similarly to what reported by Udobi *et al*.^15^, adult nes-CrT^−/y^ mice carrying the deletion of exons 5–7 present a remarkable Cr depletion restricted to the nervous system leading to a significant cognitive deficit^14,15^. Here, we performed a longitudinal evalutation of cognitive functions in nes-CrT^−/y^ mice, and we explored various autistic-like and motor behaviors in order to provide a normative portrait of conditional CTD mouse model relevant in translational perspective.

## Results

### Nervous system-specific CrT deletion leads to significant Cr deficit in the cerebral compartment

**Figure 1:**
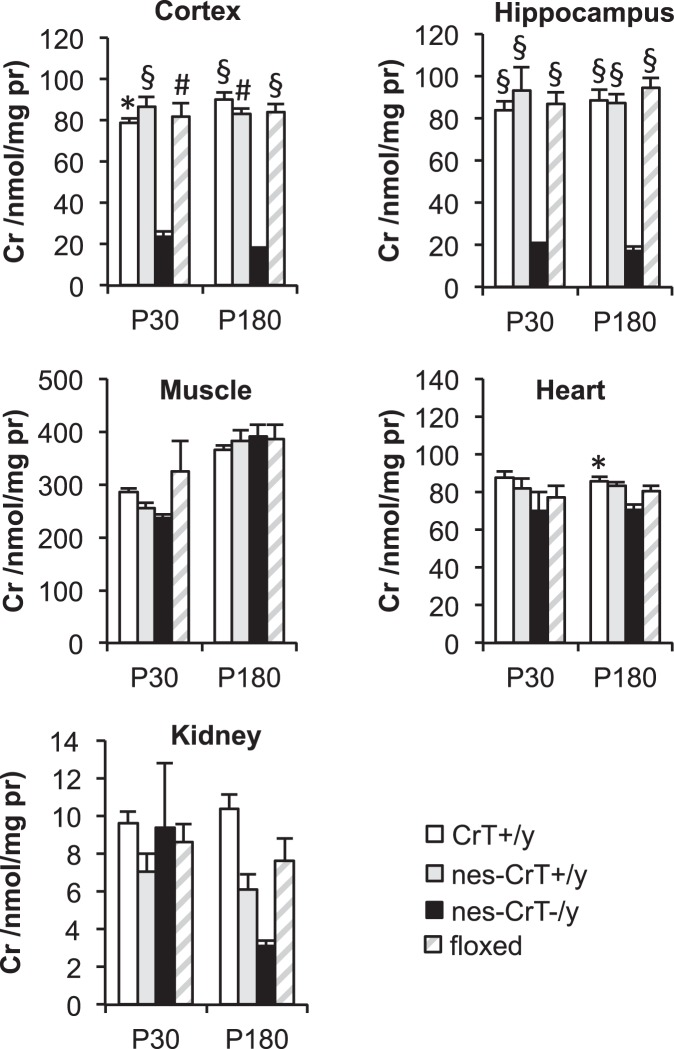
Histograms show Cr levels in CrT^+/y^, nes-CrT^+/y^, nes-CrT^−/y^ and CrT^fl/y^ animals in brain and peripheral tissues at P30 and P180 (n = 4 per tissue for all groups). Cr levels have been measured by GC/MS. At both ages tested, a reduction of Cr content was evident in the cerebral cortex (Two Way RM ANOVA on rank transformed data, genotype x tissue interaction, P30: F(12,48) = 3.452, p < 0.001; P180: F(12,48) = 3.609, p < 0.001; post hoc Holm-Sidak method, P30: p < 0.05 vs. CrT^+/y^, p < 0.001 vs. nes-CrT^+/y^, p < 0.01 vs. CrT^fl/y^; P180: p < 0.001 vs. CrT^+/y^, p < 0.01 vs. nes-CrT^+/y^, p < 0.001 vs. CrT^fl/y^) and the hippocampus of nes-CrT^−/y^ mice (p < 0.001 for all comparisons at both ages). Muscle (Two Way RM ANOVA on rank transformed data, post hoc Holm-Sidak method; P30: p = 0.890 vs. CrT^+/y^, p = 0.965 vs. nes-CrT^+/y^, p = 0.880 vs. CrT^fl/y^; P180: p = 0.985 vs. CrT^+/y^, p = 0.964 vs. nes-CrT^+/y^, p = 0.994 vs. CrT^fl/y^), heart (P30: p = 0.257 vs. CrT^+/y^, p = 0.625 vs. nes-CrT^+/y^, p = 0.969 vs. CrT^fl/y^; P180: p = 0.109 vs. nes-CrT^+/y^, p = 0.305 vs. CrT^fl/y^) and kidney (P30: p = 0.991 vs. CrT^+/y^, p = 0.971 vs. nes-CrT^+/y^, p = 0.937 vs. CrT^fl/y^; P180: p = 0.214 vs. CrT^+/y^, p = 0.742 vs. nes-CrT^+/y^, p = 0.584 vs. CrT^fl/y^) of mutant animals were preserved from Cr depletion, with the exception of the heart at P180 showing slightly decreased Cr levels with respect to CrT^+/y^ mice (p < 0.05). In addition, a Three Way ANOVA on rank transformed data analysis revealed no difference for the age factor (p = 0.301, F(1,120) = 1.080). Symbols refer to post-hoc Holm Sidak comparisons between nes-CrT^−/y^ mice and the genotype corresponding to the column on which the symbol is located: *p < 0.05, ^#^p < 0.01, ^§^p < 0.001. Error bars, s.e.m.

In order to determine the validity of our approach for tissue-specific CrT gene deletion, we quantified Cr levels in various brain regions and peripheral tissues in P30 and P180 mice using GC/MS. We found a specific decrease of Cr in the cerebral cortex and the hippocampus of mutant animals (nes-CrT^−/y^) with respect to wild-type mice (CrT^+/y^) and Cre-recombinase expressing littermates (nes-CrT^+/y^), while no significant difference was observed between CrT^+/y^ and nes-CrT^+/y^ animals^14^ (Fig. 1). At P180, lower Cr levels in the cerebellum and brainstem of nes-CrT^−/y^ mice were also observed (Fig. S1). Peripheral tissues including muscle, heart and kidney were not affected (Fig. 1). Only at P180 did we find a small difference of heart Cr content and a reduction in kidney tissue between nes-CrT^−/y^ and CrT^+/y^ mice. This is likely due to the scattered expression of Nestin promoter in heart and kidney cells. CrT^fl/y^ animals not expressing Cre recombinase did not present a hypomorph phenotype as Cr was expressed in a range similar to that of normal values (Figs 1 and S1). Thus, we performed behavioral investigations only in the other three experimental groups. Importantly, no difference in Cr levels were present between P30 and P180.

### Lower body weight in nes-CrT^–/y^ mice

Nes-CrT^−/y^ mice displayed a slightly lower body weight with respect to wild-type animals and nes-CrT^+/y^ littermates at P100, P180 and P365 (Fig. S2). Despite an age-dependent progressive growth of body mass in all experimental groups, body weight decrease in nes-CrT^−/y^ animals was approximately 10%.

### Age-dependent deterioration of cognitive functions in brain-specific mutant mice

We previously reported that nes-CrT^−/y^ mice show at P180 an impairment of both declarative and working memory^14^. To study whether and how the pathological phenotype advances in nes-CrT^−/y^ mice, we analyzed four different time points: 1. during brain development (P40), 2. in the adult age (P100), 3. in the middle age (P180), and 4. in the early aging (P365). Since mild secondary effects of Cre recombinase transgene insertion or Cre activity on selected endophenotypes have been previously suggested^19^, we also analyzed the behavior of nes-CrT^+/y^ littermates at the same time points. We report a late onset of cognitive decline in nervous system-specific nes-CrT^−/y^ mice, followed by an age-dependent increase of cognitive frailty. No alterations of learning and memory, general exploratory activity, motor function and stereotipies were detected in the Nestin (Cre) mouse line.

#### Y maze

**Figure 2:**
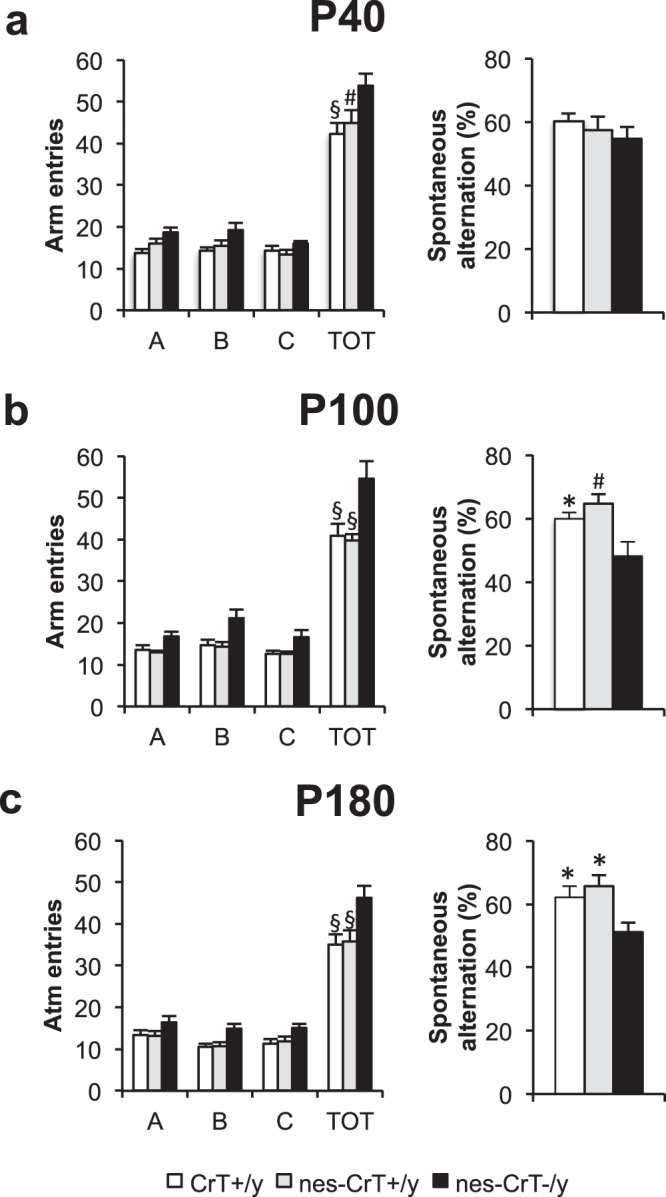
At all ages tested, an effect of genotype (Two-Way ANOVA, p < 0.001, P40: F(2,100) = 9.791, P100: F(2,100) = 16.886, P180: F(2,84) = 10.595) and an effect of arm level (Two-Way ANOVA, p < 0.001, P40: F(3,100) = 221.487, P100: F(3,100) = 203.358, P180: F(3,84) = 158.430) were detected for the number of arm entries. A post hoc Holm-Sidak method revealed an higher number of total entries (TOT) scored for nes-CrT^−/y^ mice (post hoc Holm-Sidak method, P40: p < 0.001 vs. CrT^+/y^, p < 0.01 vs. nes-CrT^+/y^; P100 and P180: p < 0.001 for both comparisons; Fig. 3a–c left side). In contrast, no difference was found in the number of entries in the single arms of the maze (A, B, C; Two-Way ANOVA, post hoc Holm-Sidak method), excluding a specific bias in arm choice. No difference in the Y maze performance was present among the three groups tested at P40 (CrT^+/y^, n = 13, nes-CrT^+/y^, n = 9, nes-CrT^−/y^, n = 6; One Way ANOVA, p = 0.417, F(2,25) = 0.906, panel a right side). In contrast, spontaneous alternation rate was significantly lower in nes-CrT^–/y^ mice compared to that recorded for CrT^+/y^ animals and nes-CrT^+/y^ littermates at P100 (One Way ANOVA, p < 0.01, F(2,25) = 6.599, post hoc Holm-Sidak method, p < 0.05 vs. CrT^+/y^, p < 0.01 vs. nes-CrT^+/y^; panel b right side) and P180 (CrT^+/y^, n = 9, nes-CrT^+/y^, n = 9, nes-CrT^−/y^, n = 6; One Way ANOVA, p < 0.05, F(2,21) = 4.766, post hoc Holm-Sidak method, p < 0.05 for both comparisons; panel c right side). Symbols refer to post-hoc Holm Sidak comparisons between nes-CrT^−/y^ mice and the genotype corresponding to the column on which the symbol is located: *p < 0.05, ^#^p < 0.01. Error bars, s.e.m.

Mutant nes-CrT^−/y^, nes-CrT^+/y^ littermates and control wild-type mice (CrT^+/y^) similarly visited the three arms of the maze (designated as A, B, C) with no bias towards a specific branch. No effect of genotype was present for the number of entries in the single arms of the maze, despite an increased total number of entries by nes-CrT^−/y^ mice (Fig. 2a). At P40, the alternation rate was not different among CrT^+/y^, nes-CrT^+/y^ and nes-CrT^−/y^ groups (Fig. 2a), whereas spatial working memory was already degraded in whole-body CrT^−/y^ mutants at this age^14^ (Fig. S3). In contrast, a deficit was noticed at P100 and P180, with a significantly lower performance of nes-CrT^−/y^ animals with respect to CrT^+/y^ age-matched mice and nes-CrT^+/y^ littermates (Fig. 2b,c). Also at these ages, the total number of entries by nes-CrT^−/y^ mice is higher compared to the two control groups, but no bias was present in the exploration of the three arms (Fig. 2b,c).

#### Object recognition test (ORT)

**Figure 3:**
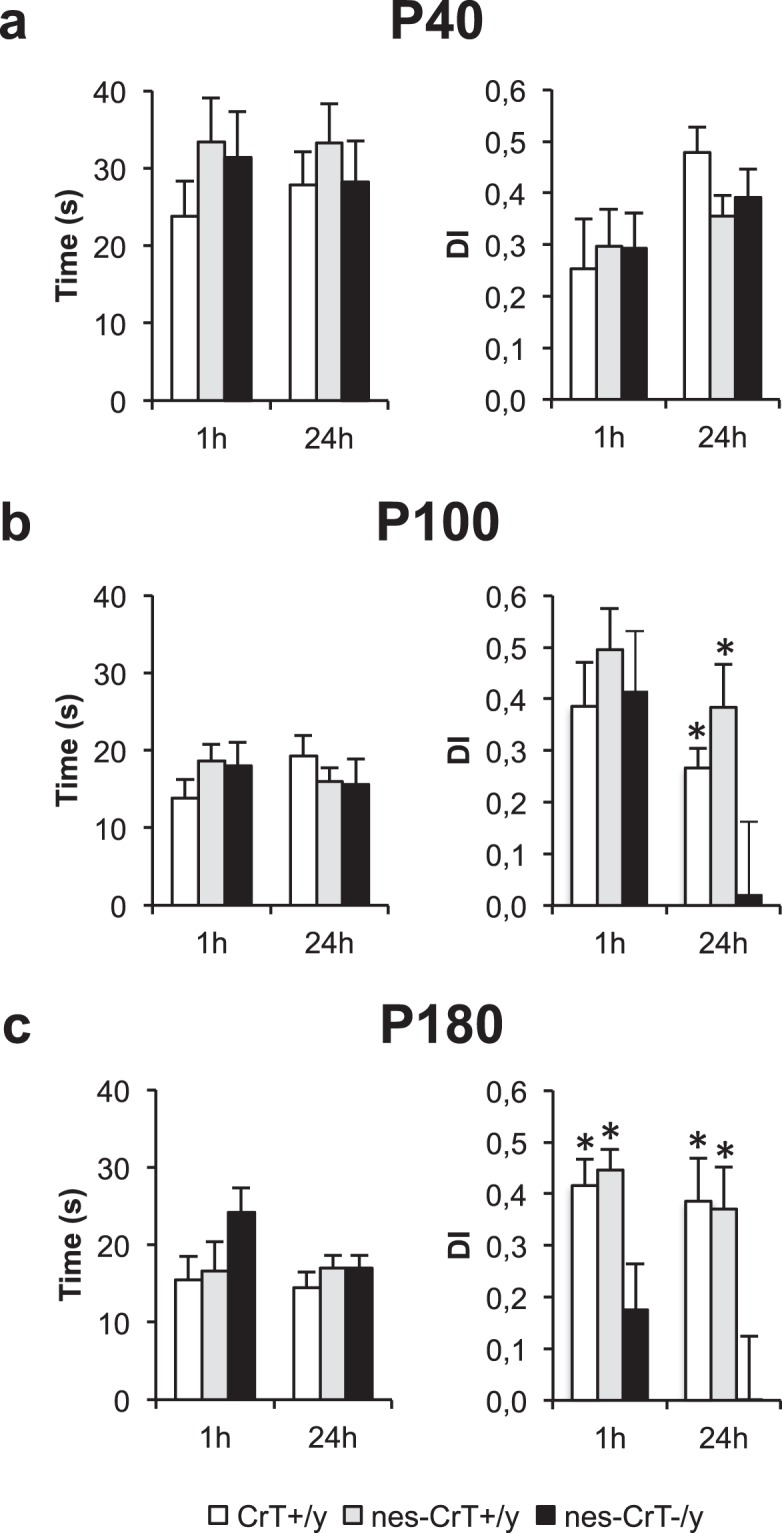
Left, diagrams describe total time of object exploration during the testing phase. (**a**,**b**,**c**) No difference was present among the different groups at all ages tested (One-Way ANOVA, P40: p = 0.372, F(2,22) = 1.036 for 1 h and p = 0.686, F(2,18) = 0.384 for 24 h; P100: p = 0.309, F(2,19) = 1.250 for 1 h and p = 0.531, F(2,21) = 0.653 for 24 h; P180: p = 0.194, F(2,19) = 1.792 for 1 h and p = 0.526, F(2,22) = 0.661 for 24 h). Right, histograms display object discrimination indexes (DIs) of CrT^+/y^, nes-CrT^+/y^ and nes-CrT^−/y^ during the testing phase performed after a delay of 1 and 24 h at different ages. (**a**) P40. The experimental groups (CrT^+/y^: n = 10, nes-CrT^+/y^: n = 8 and nes-CrT^−/y^: n = 6) can recognize the new object in the test both at 1 h (One Way ANOVA, p = 0.916, F(2,21) = 0.088) and at 24 h (p = 0.183, F(2,18) = 1.868). (**b**) P100. While the three experimental groups can recall the memory of the familiar object in the test at 1 h (One Way ANOVA, p = 0.671, F(2,19) = 0.408), a significantly lower discrimination index was found in nes-CrT^–/y^ mice (n = 6) compared to CrT^+/y^ (n = 10) and nes-CrT^+/y^ animals at 24 h (n = 9; One Way ANOVA, p < 0.05, F(2,22) = 5.064, post hoc Holm Sidak method p < 0.05 for both comparisons). (**c**) P180. A significant deficit of both short (One Way ANOVA, p < 0.05, F(2,19) = 5.662; post hoc Holm Sidak method p < 0.05 for both comparisons) and long-term memory (One Way ANOVA, p < 0.05, F(2,22) = 3.990; post hoc Holm Sidak method p < 0.05 for both comparisons) was detected in mutant mice (n = 6) compared to controls (CrT^+/y^: n = 11, nes-CrT^+/y^: n = 9). Symbols refer to post-hoc Holm Sidak comparisons between nes-CrT^−/y^ mice and the genotype corresponding to the column on which the symbol is located: *p < 0.05. Error bars, s.e.m.

Differently from what reported for whole-body CrT^−/y^ mice^14^ (Fig. S3), no difference in short- (1 h) and long-term (24 h) object recognition memory was detected at P40 in nes-CrT^−/y^ mice with respect to CrT^+/y^ and nes-CrT^+/y^ animals (Fig. 3a). The total time of object exploration during the test phase was also comparable among the three experimental groups. An impairment became apparent two months later (P100), when the discrimination index at 24 h was significantly lower in conditional mutant mice, indicating a decreased capacity to recall a familiar object (Fig. 3b). This deficit eventually affected both short and long-term memories at P180. Indeed, at this age nes-CrT^−/y^ mice displayed a marked impairment in both 1- and 24-h test compared to CrT^+/y^ and nes-CrT^+/y^ mice (Fig. 3c). At both ages, total time of exploration was still comparable among groups (Fig. 3b,c).

#### Morris water maze (MWM)

**Figure 4:**
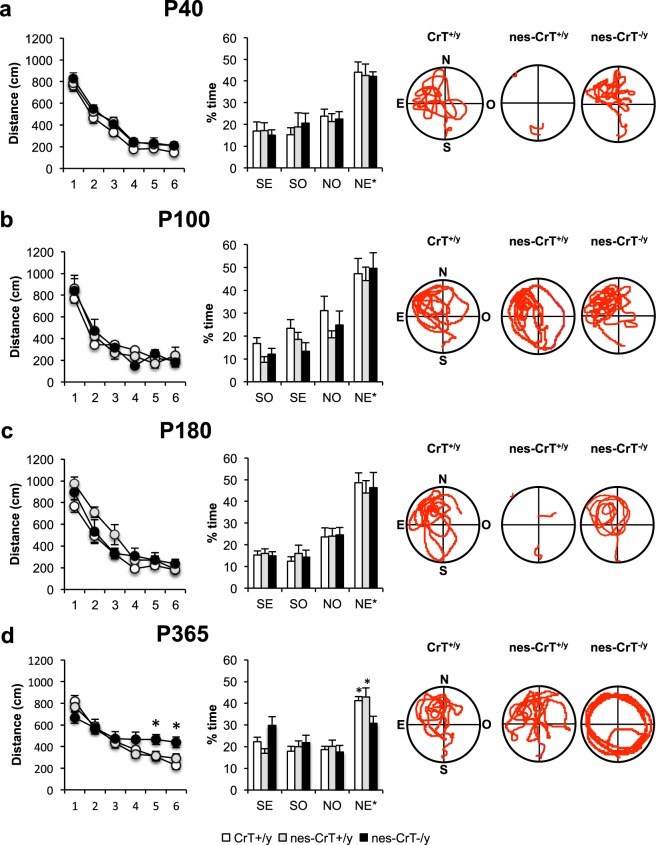
Left, learning curves for CrT^+/y^ (white), nes-CrT^+/y^ mice (grey) and nes-CrT^–/y^ (black) at P40 (**a**; CrT^+/y^: n = 10, nes-CrT^+/y^: n = 5, nes-CrT^−/y^: n = 5), P100 (**b;** CrT^+/y^: n = 7, nes-CrT^+/y^: n = 5, nes-CrT^−/y^: n = 5), P180 (**c**; CrT^+/y^: n = 9, nes-CrT^+/y^: n = 6, nes-CrT^−/y^: n = 5) and P365 (**d**; CrT^+/y^: n = 12, nes-CrT^+/y^: n = 14, nes-CrT^−/y^: n = 12). No significant difference was detected along the training phase at P40 (Two way RM ANOVA, interaction genotype x day p = 0.999, F(10,85) = 0.127), P100 (interaction genotype x day p = 0.464, F(10,70) = 0.986) and P180 (Two way RM ANOVA on rank transformed data, interaction genotype x day p = 0.748, F(10,85) = 0.671). In contrast, 1-year old nes-CrT^−/y^ animals were poorer learners with respect to control groups, with a significantly longer distance covered at day 5 and 6 of training (Two way RM ANOVA, interaction genotype x day p < 0.05, F(10, 175) = 2.334; post hoc Holm-Sidak method, p < 0.05 for all comparisons at day 5, p < 0.01 vs. CrT^+/y^, p < 0.05 vs. nes-CrT^+/y^ at day 6). Right, histograms showing the mean time percentage spent in the four quadrants during the probe trial. No significant difference among the three groups was present at P40 (**a**; Two way RM ANOVA, interaction genotype x quadrant p = 0.985, F(6,51) = 0.167), P100 (**b**; interaction genotype x quadrant p = 0.856, F(6,42) = 0.428) and P180 (**c;** interaction genotype x quadrant p = 0.985, F(6,51) = 0.166). At all ages, CrT^+/y^, nes-CrT^+/y^, nes-CrT^–/y^ spent significantly more time in the NE* target quadrant (Two way RM ANOVA, post hoc Holm Sidak method, p < 0.05 for all comparisons). At P365 (**d**), a Two-Way RM ANOVA detected a significant interaction genotype x quadrant (p < 0.05, F(6,105) = 2.534): post hoc Holm-Sidak multiple comparisons revealed that nes-CrT^−/y^ mice did not show any preference for the target quadrant (p = 0.296 NE* vs. SO, p = 0.850 NE* vs SE, p = 0.060 NE* vs. NO), while CrT^+/y^ and nes-CrT^+/y^ spent significantly more time in the NE* target quadrant (p < 0.01 for all comparisons in CrT^+/y^, p < 0.001 for all comparisons in nes-CrT^+/y^). The percentage of time spent in the target quadrant was shorter in nes-CrT^−/y^ mice than in the other two control groups (p < 0.05 for both comparisons). Representative examples of the swimming path during the probe session for a CrT^+/y^, a nes-CrT^+/y^ and a nes-CrT^−/y^ mouse are also depicted. Symbols refer to post-hoc Holm Sidak comparisons between nes-CrT^−/y^ mice and the genotype corresponding to the column on which the symbol is located: *p < 0.05. Error bars, s.e.m.

No difference was observed in the MWM performance of CrT^+/y^, nes-CrT^+/y^ and nes-CrT^−/y^ mice at P40, P100 and P180, with conditional mutant animals being able to learn the task during the training phase and to remember the position of the platform in the probe trial as well as control mice (Fig. 4a–c). In contrast, performance of whole-body CrT^−/y^ animals was scant already at P40^14^ (Fig. S3). At 1-year of age, however, nes-CrT^−/y^ mice showed a poorer performance and the distance covered by mutant animals to locate the platform was significantly longer compared to that recorded for age-matched CrT^+/y^ and nes-CrT^−/y^ animals at days 5 and 6 of training (Fig. 4d, left). The probe test confirmed the presence of a spatial memory impairment in the conditional mutant mouse: animals not carrying the floxed allele spent significantly longer time in the target quadrant whereas nes-CrT^−/y^ mice did not recall the location of the platform and equivalently explored the different quadrants (Fig. 4c, right). As expected, no difference was detected with respect to mean swimming speed during any of the six training days (Fig. S4), demonstrating that motor functions are largely preserved in the nervous system-specific CrT murine model.

### Anxiety levels and motor activity in conditional mutant mice

We also evaluated general activity and anxiety-related behavior of CrT^+/y^ mice, nes-CrT^+/y^ and nes-CrT^−/y^ in the open field arena. All animals seemed to remain in the peripheral region of the arena for a significantly longer time length; indeed, nes-CrT^−/y^ mutant mice spent a comparable amount of time in the central and peripheral portion of the square-shaped maze to that recorded for CrT^+/y^ and nes-CrT^+/y^ animals (Fig. S5). No difference was detected neither in motion speed nor total distance moved at P40 and P180, regardless of decreased observed activity in nes-CrT^+/y^ at P100 (Fig. S4). These data are in line with the lack of alterations in this test previously observed in the ubiquitary CrT mutant^14^. To evaluate motor function in non-aversive environment, we also longitudinally investigated home-cage-locomotor activity in this mouse model. We found that nes-CrT^−/y^ mice are significantly more active than the CrT^+/y^ and the nes-CrT^+/y^ group at P100. More specifically, nes-CrT^−/y^ mice showed increased horizontal activity during the night period (Fig. S6). In agreement with the data obtained with the open field test, no effect of genotype was observed at P40 and P180 (Fig. S6). Finally, we performed a direct measurement of muscle function using the conventional grip strength test in adult animals. Nes-CrT^−/y^ mice displayed a forelimb strength totally comparable to wild-type and nes-CrT^+/y^ animals (Fig. S6).

### Absence of pathological repetitive and stereotyped behavior in nes-CrT^–/y^ mice

We recently reported that ubiquitary CrT^−/y^ mutant animals exhibit increased repetitive and stereotyped behavior^14^. Thus, we examined the performance of CrT^+/y^ mice, nes-CrT^+/y^ and nes-CrT^–/y^ in the rotarod test at P40, P100, P180 and P365, and the amount of self-grooming at P180 and P365^20,21^. Despite a slight trend towards decreased motor performance, nes-CrT^–/y^ mutant mice did not differ from CrT^+/y^ and nes-CrT^+/y^ mice in the rotarod task, with a similar fall latency at all ages tested (Fig. S8a). The same was true for the self-grooming in that no difference was present in the time CrT^+/y^, nes-CrT^+/y^ and nes-CrT^–/y^ spent grooming themselves (Fig. S8b). These results suggest that the presence of autistic-like traits in the mouse model could be related to extraneural Cr deficiency.

## Discussion

We have recently generated a new murine model of human CrT deficiency carrying a nervous system-specific deletion in the murine ortholog of CrT gene^14^. In agreement with previous works^13–15^, we report that Cr nervous system-specific depletion causes a significant impairment of declarative and spatial working memory.

This is the first longitudinal analysis of behavioral deficit in a conditional CrT mouse model, showing a late onset of cognitive symptoms associated with progressive learning and memory deterioration which further culminates in one-year old animals as a broad spectrum impairment of declarative, spatial and working memory. Our MWM data are not entirely consistent with recent work suggestive that spatial learning and memory deficits could already be present at P60–90 in brain-specific CrT knockout mice^15^. However, the authors found that P60–90 CrT mutants are also impaired in the cued version of the MWM which tests mouse motivation to reach the platform without involving spatial memory. Thus, the differences between our results and the data obtained by Udobi *et al*. could be due to differences in the motivational aspects between the MWM variants. The significant alterations of performances in learning and memory tests shown by nes-CrT^−/y^ mice mirrored a deficit in cognitive abilities: i) motor abilities and muscle strength were preserved in conditional mutant mice, and ii) the analysis of activity in the central and peripheral portion of the open field revealed that nes-CrT^−/y^ animals exhibit anxiety levels and vulnerability to stress in the range of normal values. Despite the increased locomotor activity of nes-CrT^−/y^ mice (see also^15^), it is unlikely that hyperactivity might account for the cognitive deficits observed. Female CrT^+/−^ mice, indeed, are hyperactive without showing any deficit in object recognition or fear conditioning test^22^.

Moreover, we observed that the endophenotype of nes-CrT^−/y^ mice does not include behavioral traits related to autism spectrum disorders, such as rituals and stereotypies. Altogether, these results indicate that Cr depletion in the nervous system is a pivotal cause of the CTD pathological phenotype, in particular with regard to the cognitive domain but extraneural factors also play a role. Future studies will allow to elucidate the possible participation of peripheral metabolic alterations, blood biochemical milieu, immune abnormalities and intestinal microbiome in the etiology of CTD.

Nes-CrT^−/y^ mice also showed a significant decrease in body weight with respect to wild-type and nes-CrT^+/y^ animals, suggesting an alteration in the neural mechanisms controlling body weight. The size of body weight reduction, however, is relatively modest and age-dependent weight gain is still present in the conditional mouse model. Further analyses will be needed to check food intake, body composition, whole-body metabolism and hypothalamic neuronal activity in nes-CrT^−/y^ mice^23,24^, in order to identify metabolic defects underlying the pathological phenotype in conditional mutant mice.

Cr reduction observed in the brain of nes-CrT^−/y^ mice overlapped with the levels of Cr that characterize the brain of whole-body CrT knockout mice carrying the deletion of exons 5–7^11,14^ and CTD patients^25^. Cr deficiency was apparent in cerebral cortex, hippocampus, cerebellum and braistem of nes-CrT^−/y^ mice, i.e., brain regions critically involved in cognitive and behavioral defects displayed by patients. In contrast, Cr levels were completely preserved in the skeletal muscle as observed in CTD patients^26,27^. This result confirmed the restricted disruption of CrT gene in neural circuits, highlighting that a local CrT loss of function is sufficient to induce a dysregulation of Cr levels in the brain. Consistent with previous data^14^, the cognitive deterioration of nes-CrT^−/y^ animals was not accompanied by a reduction of Cr levels in the brain tissue, highlighting that the chronic deficit of Cr is the main event triggering cellular and molecular mechanisms that bring about the progressive decline of brain function.

In conclusion, our study provides data supporting the use of nes-CrT mice as a novel model for studying nervous-system specific mechanisms of CTD. Although displaying the impairment of both declarative and working memory in the adult age, the conditional CrT mice did not reproduce the early pathological phenotype and the autistic-like traits of CTD patients, highlighting that they do not represent a comprehensive tool for preclinical evaluation of potential CTD treatments. Nevertheless, we showed that extraneural Cr loss might modulate the phenotype of CrT mutants, making the use of nervous-system specific models like nes-Crt mice important tools to understand the pathogenetic mechanisms underlying neurological deficits induced by Cr deficiency.

## Methods

### Animals

Male mice were used for this study. CrT^+/y^, nes-CrT^+/y^ and nes-CrT^−/y^ mice on a C57BL/6 J background were generated as reported previously^12,14^. CrT^+/fl^ females were crossed with nestin::Cre male mice^18^. Animals with four genotypes were tested: wild-type animals (CrT^+/y^), mice expressing Cre-recombinase but not carrying the floxed allele (nes-CrT^+/y^), mice carrying the brain specific deletion of CrT (nes-CrT^−/y^) and mice expressing the floxed allele but not Cre-recombinase (CrT^fl/y^). Animals in each experimental group came from different litters, with a minimum of three litters for all groups to prevent litter effects. Genotyping has been performed by standard PCR as previously described^14^. All experiments were carried out in accordance with the European Directive of 22 September 2010 (2010/63/UE) and were approved by the Italian Ministry of Health (authorization number 259/2016-PR). The experiments conform with the ARRIVE (Animal Research: Reporting of *In Vivo* Experiments) guidelines and the ARRIVE Checklist is available in Supplementary Material.

### Behavioral testing

Behavioral analysis was performed as follows: open field (1 day), object recognition test (ORT) at 1 h (1 day), ORT at 24 h (3 days), Y maze (1 day), Morris water maze (MWM, 7 days), rotarod and grip strength (1 day), and self-grooming (1 day). Open field, ORT, Y maze, rotarod, grip strength and self-grooming were longitudinally perfomed in the same animals. In contrast, for MWM we used separate groups of animals at the different ages tested. In order to reduce the circadian effects, behavioral tests were performed during the same time interval each day (14:00–18:00 h; light phase). The analysis was conducted in blind with respect to the mouse genotype. Mice were weighed at the end of each experimental schedule. Behavioral tests were performed as previously described^12,14,28^. For further details, refer to Supplementary Material.

### Biochemical analysis

For Cr assay, mouse tissues were frozen on dry ice and stored at −80 °C. Biochemical analysis was performed as described^29^. See Supplementary material for more details.

### Statistical analysis

Statistical analysis was performed using SigmaPlot 12.0 Software. The significance of factorial effects and differences among more than two groups were evaluated with ANOVA/RM ANOVA followed by post hoc Holm-Sidak comparisons. Rank transformation was exploited for data not normally distributed. The level of significance was p < 0.05. P-values and F-values are reported in Figure Legends.

### Ethics approval

All experiments were carried out in accordance with the European Directive of 22 September 2010 (2010/63/UE) and were approved by the Italian Ministry of Health (authorization number 259/2016-PR).

## Supplementary information

Supplementary info

## Data Availability

The datasets generated during the current study are available from the corresponding author on reasonable request.
