# Supplementary material for: A Nervous System-Specific Model of Creatine Transporter Deficiency Recapitulates the Cognitive Endophenotype of the Disease: a Longitudinal Study
Source: Sci Rep. 2019 Jan 11;9:62. doi: 10.1038/s41598-018-37303-1 (PMC6329805; doi:10.1038/s41598-018-37303-1)
Supplement: Supplementary file 1 — Supplementary info [file 41598_2018_37303_MOESM1_ESM.pdf]

A Nervous System-Specific Model of Creatine Transporter Deficiency Recapitulates the Cognitive Endophenotype of the Disease: a Longitudinal Study

Angelo Molinaro[1,2], Maria Grazia Alessandrì[3], Elena Putignano[1], Vincenzo Leuzzi[4], Giovanni Cioni[3,5], Laura Baroncelli[1,3*#], Tommaso Pizzorusso[1,2#]

[1] Institute of Neuroscience, National Research Council (CNR), I-56124, Pisa, Italy

[2] Department of Neuroscience, Psychology, Drug Research and Child Health NEUROFARBA, University of Florence, I-50135, Florence, Italy

[3] Department of Developmental Neuroscience, IRCCS Stella Maris Foundation, I-56128 Pisa, Italy

[4] Department of Paediatrics, Child Neurology and Psychiatry, Sapienza University of Rome, I-00184, Rome, Italy

[5] Department of Clinical and Experimental Medicine, University of Pisa, I-56126, Pisa, Italy

[#]These authors equally contributed to this work.

Corresponding author:

Laura Baroncelli

Institute of Neuroscience, National Research Council (CNR)

via Moruzzi 1, Pisa I-56124, Italy.

Email: baroncelli@in.cnr.it

Tel: +390 503 153199 Fax: +390 503 153220

Methods

Animals

As CrT deficiency is an X-linked pathology, male mice were selected for this study. CrT$^{+/y}$, nes-CrT$^{+/y}$ and nes-CrT$^{-/y}$ mice on a C57BL/6J background were generated as described previously[1,2]. CrT$^{+/fl}$ females were crossed with nestin::Cre male mice[3] to generate a mouse line carrying the floxed CrT and nestin::Cre alleles. The Nestin promoter is expressed in the central and peripheral nervous system, including neurons, astrocytes, blood-brain barrier endothelial cells, neuronal and glial cell precursors, while only few isolated kidney and heart cells were reported to show Cre recombinase activity in non-neural tissues[3]. Moreover, Nestin expression is present in nervous tissue by embryonic day 11 (https://www.jax.org/strain/003771). Thus, the pattern of CrT deletion in this mouse more closely mimics the genetic cause of the human CTD disease with respect to the conditional model with selective postnatal deletion in excitatory forebrain neurons driven by CaMKIIα promoter[4], still avoiding the muscle alterations observed in the whole-body knockout[1,2,5,6]. Mice with four genotypes were used as experimental animals: wild-type animals (CrT$^{+/y}$), mice expressing Cre-recombinase but not carrying the floxed allele (nes-CrT$^{+/y}$), mice carrying the brain specific deletion of CrT (nes-CrT$^{-/y}$) and mice expressing the floxed allele but not Cre-recombinase (CrT$^{fl/y}$). These genotypes were obtained in the same litters. CrT$^{fl/y}$ mice did not display a hypomorphic phenotype with no difference in Cr levels compared to CrT$^{+/y}$ and nes-CrT$^{+/y}$ animals at both ages tested. Thus, we performed behavioral investigations only in the other three experimental groups. The number of animals required for this work has been estimated using two main criteria: i) the possibility to obtain reliable and reproducible results, ii) the reduction to a minimum number of the amount of sacrificed animals. To this purpose, the same animals have be tested in different behavioural tasks. In addition, at the end of experiments, tissues have been taken for biochemical analysis. Each individual mouse is considered an experimental unit. Animals were maintained at 22°C (humidity 40-60%) under a 12-h light–dark cycle (lights turn on at 7 AM). Housing rooms have an average lighting intensity of 1.2 cd/m2 and are equipped with automatic ventilation and suction control system. Animals are housed in Standard Cages Mod. 1144B, 1264C Eurostandard Type II, 1284L Eurostandard Type II L or 1290D Eurostandard Type III of the TECNIPLAST company (depending on the number of animals, according to Table 1.1 on the minimum housing dimensions attached to the Italian decree 26/2014 on animal experimental research) equipped with wooden litter, with urine and dewatering power, decontaminated and dust-free. The litter is changed and

cages are thoroughly cleaned twice a week. Animals are housed in groups of the same sex and genotype. Litters are weaned and separated by mothers when puppies reach P28-30. Cages that house nesting and young animals are equipped with special materials for nest building to provide conditions for a protective environment and temperature rise. Food (4RF25 GLP Certificate, Mucedola) and water were available ad libitum. All experiments were carried out in accordance with the European Directive of 22 September 2010 (2010/63/UE) and were approved by the Italian Ministry of Health (authorization number 259/2016-PR).

Detection of Slc6a8 mutation by PCR

Genomic DNA was isolated from mouse tail using a kit, and the protocol suggested by the manufacturer (DNeasy Blood & Tissue Kit, Qiagen, USA). DNA was amplified for CrT mutant and wild-type (WT) allele using a standard PCR protocol with the following primers: F:AGGTTTCCTCAGGTTATAGAGA; R:CCCTAGGTGTATCTAACATCT; R1: TCGTGGTATCGTTATGCGCC. Primers for Cre recombinase expression were: F: AACGCACTGATTTCGACC; R: CAACACCATTTTTTCTGACCC. For PCR amplification we used 300 ng of DNA in a 25 μL reaction volume containing 0.2 mM of each dNTP, 2 μM of F primer, 1 μM of R, 1 μM of R1 primer and 0.5 U/μL Red Taq DNA polymerase (Sigma-Aldrich, Italy). The PCR conditions were as follows: 94°C for 4 min followed by 37 cycles at 94°C for 30 s, 58°C for 30 s, 72°C for 40 s and a final extension at 72°C for 7 min. Amplicons were separated using 2% agarose gel and visualized under UV light after staining with Green Gel Plus (Fisher Molecular Biology, Rome, Italy). Amplicon sizes were: WT allele = 462 bp; mutant allele = 371 bp; Cre allele = 310 bp.

Behavioral testing

The testing order for behavioral assessment performed in the same animals consisted of: open field (1 day duration), object recognition test (ORT) at 1h (1 day), ORT at 24h (3 days), Y maze (1 day), Morris water maze (MWM) with hidden platform (7 days), rotarod and grip strength (1 day), and self-grooming (1 day). Mice were tested in one task at a time with the next behavioral test starting at least 1 days after the completion of the previous one. While open field, ORT, Y maze, rotarod, grip strength and self-grooming were longitudinally administered to the same animals, MWM was performed in separate groups of animals at the different ages tested. In order to reduce the circadian effects, behavioral tests were performed during the same time interval each day (14:00–18:00h;

light phase). All behavioral tests were conducted in blind with respect to the genotype of animals. Animals not performing the task were excluded from the analysis. Mice were weighed at the end of experiments.

Open field and object recognition test (ORT)

The apparatus consisted of a square arena (60 × 60 × 30 cm) constructed in poly(vinyl chloride) with black walls and a white floor. Mice received one session of 10-min duration in the empty arena to habituate them to the apparatus and test room. Animal position was continuously recorded by a video tracking system (Noldus Ethovision XT). In the recording software an area corresponding to the center of the arena (a central square 30 × 30 cm), and a peripheral region (corresponding to the remaining portion of the arena) were defined. The total movement of the animal and the time spent in the center or in the periphery area were automatically computed. Mouse activity during this habituation session was analyzed for evaluating the behavior in the open field arena. The ORT consisted of two phases: sample and testing phase. During the sample phase, two identical objects were placed in diagonally opposite corners of the arena, approximately 6 cm from the walls, and mice were allowed 10 min to explore the objects, then they were returned to their cage. The objects to be discriminated were made of plastic, metal, or glass material and were too heavy to be displaced by the mice. The testing phase was performed either 1h or 24h after the sample phase. One of the two familiar objects was replaced with a new one, while the other object was replaced by an identical copy. The objects were placed in the same locations as the previous ones. The mice were allowed to explore objects for 5 min. To avoid possible preferences for one of two objects, the choice of the new and old object and the position of the new one were randomized among animals. The amount of time spent exploring each object (nose sniffing and head orientation within <1.0 cm) was recorded and evaluated by the experimenter blind to the mouse genotype. Arena and objects were cleaned with 10% ethanol between trials to stop the build-up of olfactory cues. Mice exploring the two objects for less than 10 s during the sample phase were excluded from testing. A discrimination index was computed as DI = (T new - T old)/(T new + T old), where T new is the time spent exploring the new object, and T old is the time spent exploring the old one.

Y maze spontaneous alternation

We used a Y-shaped maze with three symmetrical grey solid plastic arms at a 120-degree angle (26 cm length, 10 cm width, and 15 cm height). Mice were placed in the center of the maze and allowed to freely explore the maze for 8 minutes. The apparatus was cleaned with 10% ethanol between trials to avoid the build-up of odor traces. All sessions were video-recorded (Noldus Ethovision XT) for offline blind analysis. The arm entry was defined as all four limbs within the arm. A triad was defined as a set of three arm entries, when each entry was to a different arm of the maze. The number of arm entries and the number of triads were recorded in order to calculate the alternation percentage (generated by dividing the number of triads by the number of possible alternations and then multiplying by 100).

Morris water maze

Mice were trained for four trials per day and for a total of 7 days in a circular water tank, made from grey polypropylene (diameter, 120 cm; height, 40 cm), filled to a depth of 25 cm with water (23°C) rendered opaque by the addition of a non-toxic white paint. Four positions around the edge of the tank were arbitrarily designated North (N), South (S), East (E), and West (W), which provided four alternative start positions and also defined the division of the tank into four quadrants, i.e., NE, SE, SW, and NW. A square clear Perspex escape platform (11 × 11 cm) was submerged 0.5 cm below the water surface and placed at the midpoint of one of the four quadrants. The hidden platform remained in the same quadrant during training, while the start positions (N, S, E, or W) were randomized across trials. The pool was situated in a room containing extra-maze cues that provide specific visual reference points for locating the submerged platform. Mice were allowed up to 60 s to locate the escape platform, and their swimming paths were automatically recorded by the Noldus Ethovision system. If the mouse failed to reach the platform within 60 s, the trial was terminated, and the mouse was guided onto the platform for 15 s. On the last trial of the last training day, mice received a probe trial, during which the escape platform was removed from the tank and the swimming paths were recorded over 60 s while mice searched for the missing platform. The swimming paths were recorded and analyzed with the Noldus Ethovision system.

Measurement of spontaneous locomotor activity

Opto M3 multi-channel activity monitors (Columbus Instruments, OH, USA) were used to quantify spontaneous horizontal activity of animals. Monitors were placed in the colony

area, and testing was conducted in the same conditions of animal facility housing. All measurements were performed from 6:00 P.M. to 6:00 A.M. (dark phase) and to 6:00 A.M. to 6:00 P.M. (light phase), using animals maintained on a 12 hr light/dark cycle from 6:00 A.M. to 6:00 P.M. Individual mice were placed in 33 × 15 × 13-cm (length × width × height) clear plastic cages for 24h and total distance travelled was calculated from infrared beam breaks by determining activity at 1-min intervals. Horizontal activity was measured by the sequential breaking of infrared beams, 2.54 cm on center, in the horizontal plane of the x axis.

Rotarod and grip strength

Motor coordination and abilities were assessed using the rotarod test. Animals were placed on a drum with increasing rotation speed from 4 to 40 rpm. The time spent on the drum was recorded by an automated unit, which stops as the mouse fall. Motor abilities were assessed by conducting the test for four consecutive times with an interval of 5 min in the same day. In the grip strength test a peak amplifier automatically measures the peak pull-force achieved by animals' forelimbs.

Self-grooming

Mice were scored for spontaneous grooming behaviors. Each mouse was placed individually into a clean, empty, standard mouse cage (27 length × 20 cm wide × 15 cm high) without bedding. Animal behaviors were videotaped for 20 min. After a 10-min habituation period in the test cage, each mouse was scored with a stopwatch for 10 min for cumulative time spent grooming all body regions.

Biochemical analysis

For Cr assay, mouse tissues, immediately frozen on dry ice and stored at -80°C until the analysis, were homogenized in 0.7 ml PBS buffer (Sigma-Aldrich, Italy) at 4°C using a ultrasonic disruptor (Microson Heat System, NY, USA) for brain or a glass manual homogenizer (VWR, Italy) for kidney, heart and muscle. After centrifugation (600 × g for 10 min at 4°C) an aliquot of the homogenate (50 µl) was assayed for protein content and the supernatant used for Cr assay as previously described[7]. Briefly, 50 µl of saturated sodium hydrogen carbonate and 50 µl of a mixture containing 2- phenylbutyric acid (I.S.) in toluene (6.09 mmol/l; Sigma-Aldrich, Italy) were added to 200 µl of homogenate. After adding 1 ml of toluene and 50 µl of hexafluoro-2,4-pentanedione (Sigma-Aldrich, Italy) to

form bis-trifluoromethyl- pyrimidine derivatives, the mixture was stirred overnight at 80°C. The organic layer was centrifuged, dried under nitrogen and 2 µl of the residue derivatized at room temperature with 100 µl of BSTFA+TMCS (Sigma-Aldrich, Italy) injected into the Gas Chromatography/Mass Spectrometry (GC/MS) instrument. GC analyses were performed using an Agilent 6890N GC equipped with an HP5MS capillary column (0.25 mm × 30 m, film thickness 0.25 ìm) and an Agilent mass spectrometer 5973N (Agilent Technologies, Italy). The mass spectrometer was set in EI- single ion monitoring mode (SIM). The ions with m/z of 192 for I.S. and 258 for Cr were used for calculation of the metabolites, using standard curves ranging 5–90 µmol/L. Data were processed by the G1701DA MSD ChemStation software. All the aqueous solutions were prepared using ultrapure water produced by a Millipore system.

References

1. Baroncelli, L. et al. A novel mouse model of creatine transporter deficiency. F1000Res. 3, 228 (2014).
2. Baroncelli, L. et al. A mouse model for creatine transporter deficiency reveals early onset cognitive impairment and neuropathology associated with brain aging. Hum. Mol. Genet. 25, 4186–4200 (2016).
3. Tronche, F. et al. Disruption of the glucocorticoid receptor gene in the nervous system results in reduced anxiety. Nat. Genet. 23, 99–103 (1999).
4. Kurosawa, Y. et al. Cyclocreatine treatment improves cognition in mice with creatine transporter deficiency. J. Clin. Invest. 122, 2837–2846 (2012).
5. Skelton, M. R. et al. Creatine transporter (CrT; Slc6a8) knockout mice as a model of human CrT deficiency. PLoS One 6, e16187 (2011).
6. Russell, A. P. et al. Creatine transporter (SLC6A8) knockout mice display an increased capacity for in vitro creatine biosynthesis in skeletal muscle. Front. Physiol. 5, 314 (2014).
7. Alessandrì, M. G., Celati, L., Battini, R., Casarano, M. & Cioni, G. Gas chromatography/mass spectrometry assay for arginine: glycine-amidinotransferase deficiency. Anal. Biochem. 343, 356–358 (2005).

Fig. S1

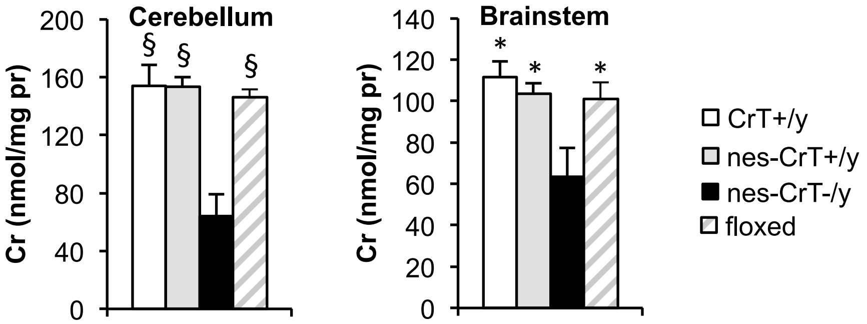

Fig. S1. Histograms show Cr levels in $CrT^{+/y}$, nes-$CrT^{+/y}$, nes-$CrT^{-/y}$ and $CrT^{fl/y}$ animals in cerebellum and brainstem at P180 (n = 4 per tissue for all groups). A decrease of Cr content was evident both in the cerebellum (Two Way RM ANOVA, genotype x tissue interaction, $p < 0.05$, $F(3,12) = 5.203$; post hoc Holm-Sidak method, $p < 0.001$ for all comparisons) and the brainstem of nes-$CrT^{-/y}$ mice ($p < 0.05$ for all comparisons). Symbols refer to post-hoc Holm Sidak comparisons between nes-$CrT^{-/y}$ mice and the genotype corresponding to the column on which the symbol is located: * $p < 0.05$, § $p < 0.001$. Error bars, s.e.m.

Fig. S2

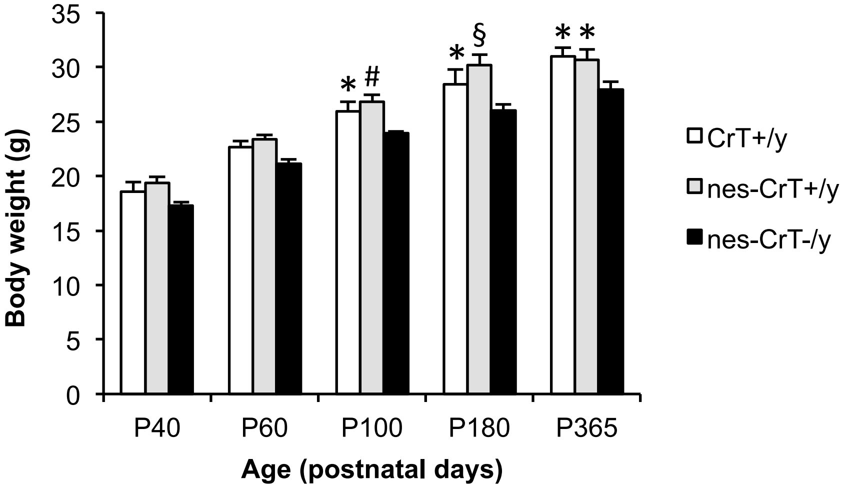

Fig. S2. The weight of nes-CrT$^{-/y}$ mice (n = 6) was significantly reduced compared to CrT$^{+/y}$ (n = 13) and nes-CrT$^{+/y}$ animals (n = 9) at P100 (Two Way ANOVA on rank transformed data, effect of genotype $p < 0.001$ and $F (2,120) = 17.854$, post hoc Holm-Sidak method, $p < 0.05$ vs. CrT$^{+/y}$, $p < 0.01$ vs. nes-CrT$^{+/y}$), P180 ($p < 0.05$ vs. CrT$^{+/y}$, $p < 0.001$ vs. nes-CrT$^{+/y}$) and P365 ($p < 0.05$ for both comparisons). No difference was detected among the three groups at P40 (Two Way ANOVA on rank transformed data, post hoc Holm-Sidak method, $p = 0.457$ vs. CrT$^{+/y}$, $p = 0.373$ vs. nes-CrT$^{+/y}$) and P60 ($p = 0.192$ vs. CrT$^{+/y}$, $p = 0.069$ vs. nes-CrT$^{+/y}$), although the body weight of mutant animals is slightly lower even at these ages with respect to the other two groups. It is worth noting that age-dependent growth of body mass was not compromised in nes-CrT$^{-/y}$ animals showing a progressive increase of their weight (Two way ANOVA on rank transformed data, $p < 0.001$ and $F (4,120) = 138.701$ for age effect, $p < 0.05$ for all age comparisons within CrT$^{+/y}$, nes-CrT$^{-/y}$ and nes-CrT$^{+/y}$ groups). Symbols refer to post-hoc Holm Sidak comparisons between nes-CrT$^{-/y}$ mice and the genotype corresponding to the column on which the symbol is located: * $p < 0.05$, # $p < 0.01$, § $p < 0.001$. Error bars, s.e.m.

Fig. S3

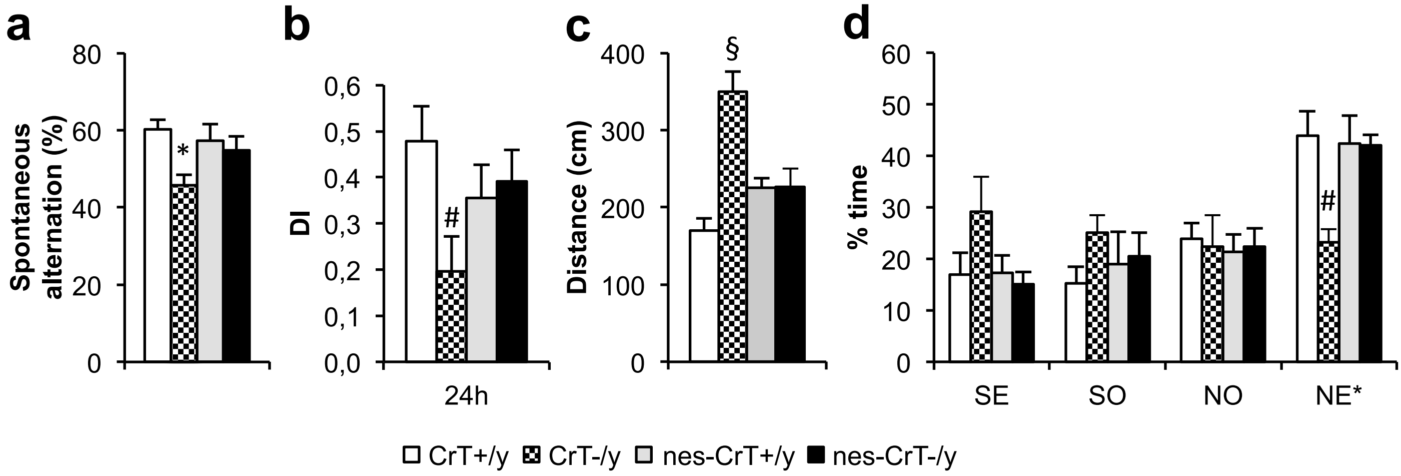

Fig. S3. The onset of cognitive decline in nervous system-specific nes-$CrT^{-/y}$ mice is temporally delayed with respect to whole body $CrT^{-/y}$ mutants. (a) A significant deterioration in Y maze performance was present in $CrT^{-/y}$ mice (n = 7) at P40 (One Way ANOVA, $p < 0.05$, $F(3,31)= 3.471$, post hoc Holm Sidak method vs. wt group, $p < 0.05$), whereas the alternation rate of nes-$CrT^{-/y}$ (n = 6) and nes-$CrT^{+/y}$ (n = 9) was in the wt range ($CrT^{+/y}$, n = 13, p = 0.454 and p = 0.498, respectively). (b) A significantly lower discrimination index was found in P40 $CrT^{-/y}$ mice (n = 10) in the ORT test at 24h (One Way ANOVA, $p < 0.01$, $F(3,27) = 4.756$, post hoc Holm Sidak method vs. wt group, $p < 0.01$) whereas age-matched nes-$CrT^{-/y}$ (n = 6) and nes-$CrT^{+/y}$ animals (n = 8) could recall the memory of the familiar object similarly to $CrT^{+/y}$ mice (n = 7; p = 0.363 and p = 0.305, respectively). (c) The histograms show the mean swiming path covered to locate the submerged platform during the last three days of MWM training for $CrT^{+/y}$ (n = 10), $CrT^{-/y}$ (n = 7), nes-$CrT^{+/y}$ (n = 5) and nes-$CrT^{-/y}$ (n = 5) groups. One Way ANOVA analysis revealed a significant effect of genotype ($p < 0.001$, $F(3,23) = 10.956$) with $CrT^{-/y}$ showing a longer distance in the training phase (post hoc Holm Sidak comparison vs. wt group $p < 0.001$). No difference was evident in the performance of nes-CrT+/y (p =0.122) and nes-CrT-/y mice (p = 0.218). (d) Histograms showing the mean time percentage spent in the four quadrants during the probe trial at P40. The percentage of time spent in the target quadrant was shorter in $CrT^{-/y}$ mice than in $CrT^{+/y}$ animals (Two Way RM ANOVA, interaction genotype x quadrant p = 0.073, $F(9,72)= 1.853$, post hoc Holm Sidak method vs. wt group, $p < 0.01$), whereas no difference was detected among the other three experimental groups (p = 0.490 for nes-$CrT^{+/y}$ group and p =0.781 for nes-$CrT^{-/y}$ group). Symbols refer to post-hoc Holm Sidak comparisons between $CrT^{+/y}$ mice and the genotype corresponding to the column on which the symbol is located: * $p < 0.05$, # $p < 0.01$, § $p < 0.001$. Error bars, s.e.m.

Fig. S4

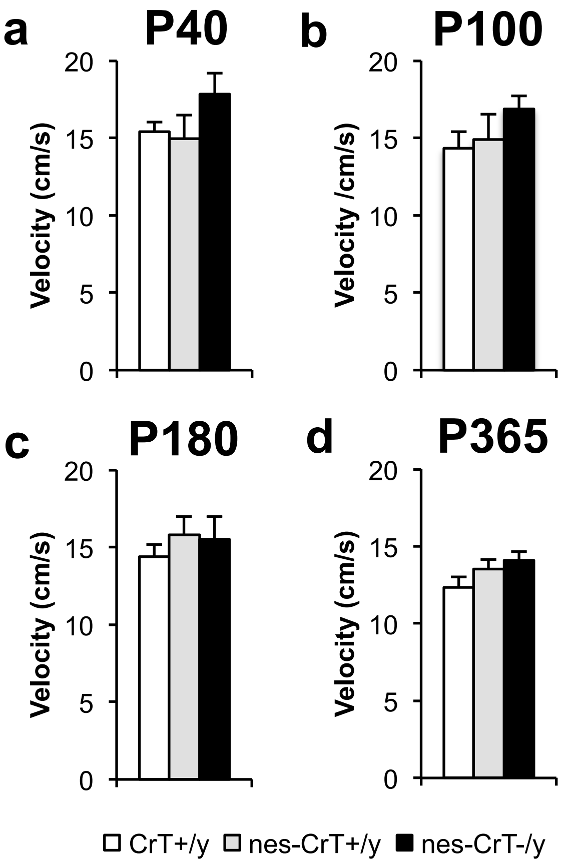

Fig. S4. Mean swimming speed measured all along the training phase of the Morris water maze for $CrT^{+/y}$, nes- $CrT^{+/y}$, and nes-$CrT^{-/y}$ animals at P40 (a; $CrT^{+/y}$: n = 10, nes-$CrT^{+/y}$: n = 5, nes-$CrT^{-/y}$: n = 5), P100 (b; $CrT^{+/y}$: n = 7, nes-$CrT^{+/y}$: n = 5, nes-$CrT^{-/y}$: n = 5), P180 (c; $CrT^{+/y}$: n = 9, nes-$CrT^{+/y}$: n = 6, nes-$CrT^{-/y}$: n = 5) and P365 (d; $CrT^{+/y}$: n = 12, nes-$CrT^{+/y}$: n = 14, nes-$CrT^{-/y}$: n = 12). At all ages tested, mutant nes-$CrT^{-/y}$ mice resulted to be good swimmers as well as control animals (One Way ANOVA, p = 0.191 and F (2,17) = 1.827 at P40, p = 0.336 and F (2,14) = 1.179 at P100, p = 0.592 and F (2,17) = 0.540 at P180, p = 0.177 and F (2,35) = 1.822 at P365). Error bars, s.e.m.

Fig. S5

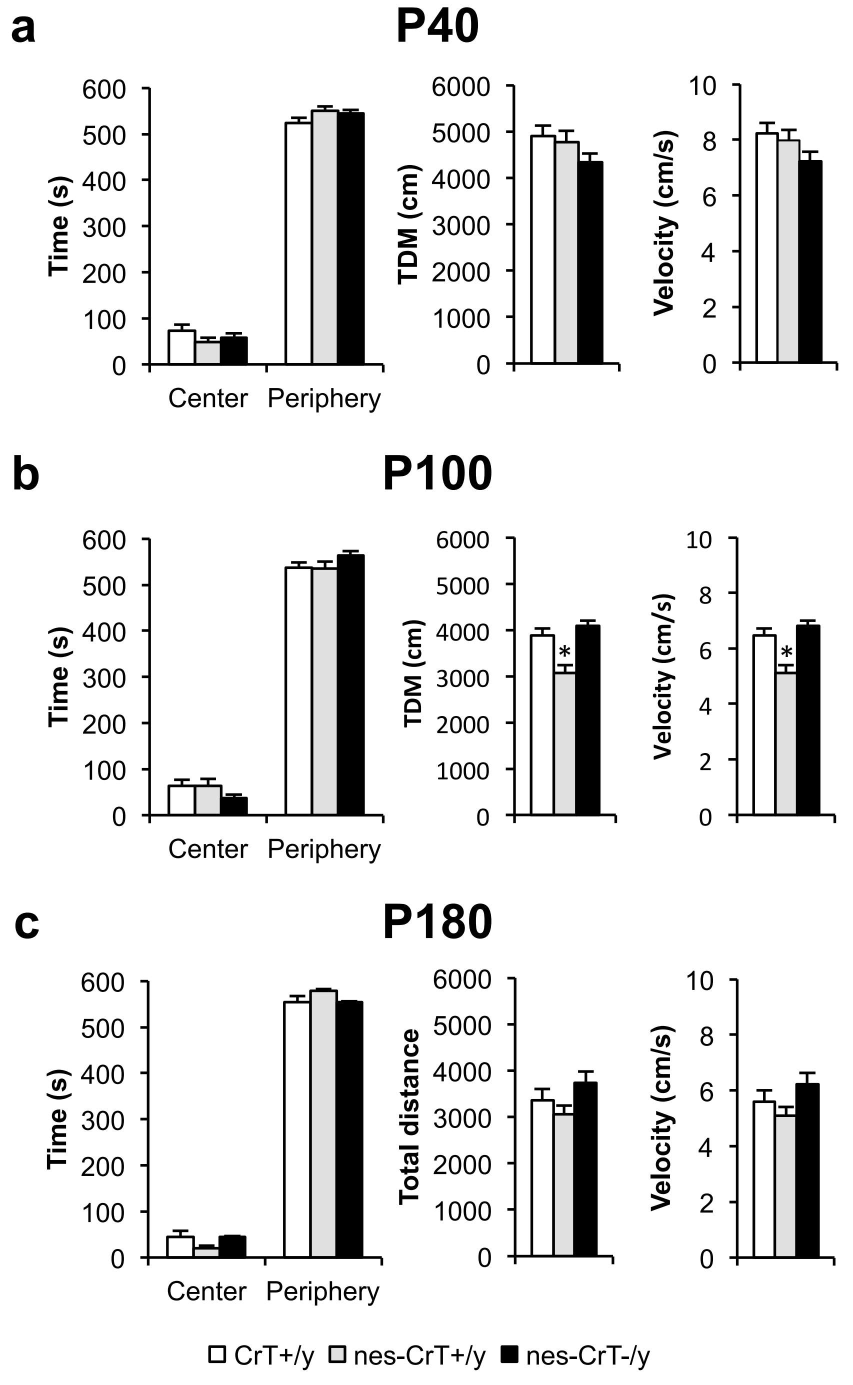

Fig. S5. Left, $CrT^{+/y}$, nes-$CrT^{+/y}$ and nes-$CrT^{-/y}$ and $CrT^{+/y}$ mice spent a comparable amount of time in the center and in the peripheral region of the open field arena. A Kruskal-Wallis One Way ANOVA on ranks analysis shows no significant effect of genotype at P40 ($CrT^{+/y}$ n = 13, nes-$CrT^{+/y}$ n = 9, nes-$CrT^{-/y}$ n = 6; p = 0.384 and H (2) = 1.917; panel a), P100 ($CrT^{+/y}$ n = 13, nes-$CrT^{+/y}$ n = 9, nes-$CrT^{-/y}$ n = 6; p = 0.162 and H (2) = 3.643; panel b) and P180 ($CrT^{+/y}$ n = 11, nes-$CrT^{+/y}$ n = 8, nes-$CrT^{-/y}$ n = 6; p = 0.588 and H (2) = 1.061; panel c). Middle and right, total distance moved and velocity did not differ between CrT conditional mutants and control animals at P40 (One Way ANOVA, p = 0.159, F (2,25) = 1.978) and P180 (One Way ANOVA, p = 0.215, F (2,22) = 1.649), while nes-$CrT^{+/y}$ mice displayed a lower speed and, consequently, a shorter path covered in the arena at P100 (Kruskal-Wallis One Way ANOVA on ranks, effect of genotype p < 0.01 and H (2) = 13.156, post hoc Dunn's method p < 0.05 for both comparisons). Symbols refer to post-hoc Holm Sidak comparisons between nes-CrT-/y mice and the genotype corresponding to the column on which the symbol is located: * p < 0.05. Error bars, s.e.m.

Fig. S6

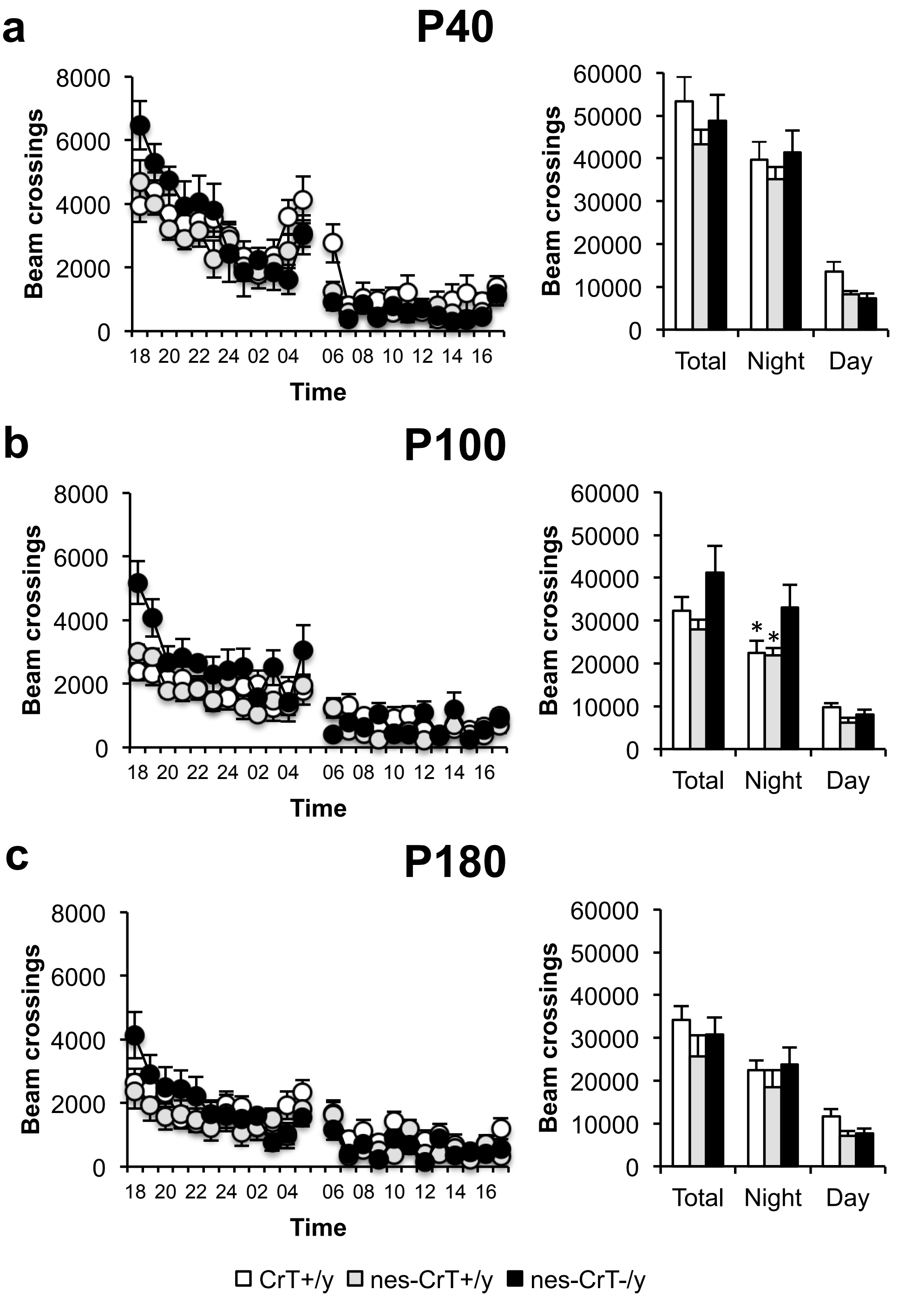

Fig. S6. Left, time course of horizontal activity of $CrT^{+/y}$ (white), nes-$CrT^{+/y}$ (grey) and nes-$CrT^{-/y}$ (black) animals during 24h at P40 (a), P100 (b) and P180 (c). Data are plotted as total number of beam crossings ± SEM in each time block of 60 min. Right, total horizontal distance travelled throughout 24h (total), and over the dark (night) or light phase (day). No significant difference was detected among the three genotypes at P40 and P180 (Two Way ANOVA on rank transformed data, $p = 0.384$, $F(2,75) = 0.970$ and $p = 0.157$, $F(2,75) = 0.478$, respectively), while nes-$CrT^{-/y}$ mice had a significant increase in motor activity at P100 in comparison to control groups during the night period (Two-Way ANOVA, effect of genotype $p < 0.05$ and $F(2,75) = 4.909$, post hoc Holm-Sidak method, $p < 0.05$ for both comparisons). Symbols refer to post-hoc Holm Sidak comparisons between nes-CrT-/y mice and the genotype corresponding to the column on which the symbol is located: * $p < 0.05$. Error bars, s.e.m.

Fig. S7

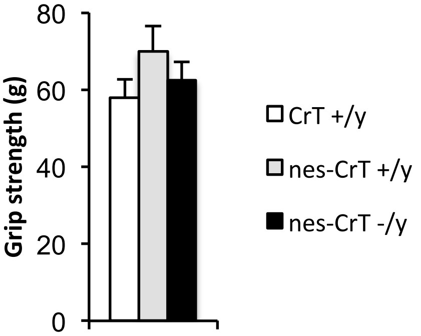

Fig. S7. The performance of nes-CrT$^{-/y}$ mice (n = 7) in the grip strength displayed no significant difference with respect to CrT$^{+/y}$ (n = 13) and nes-CrT$^{+/y}$ (n = 11) animals (One Way ANOVA, p = 0.191 and F (2,28) = 1.759). Error bars, s.e.m.

Fig. S8

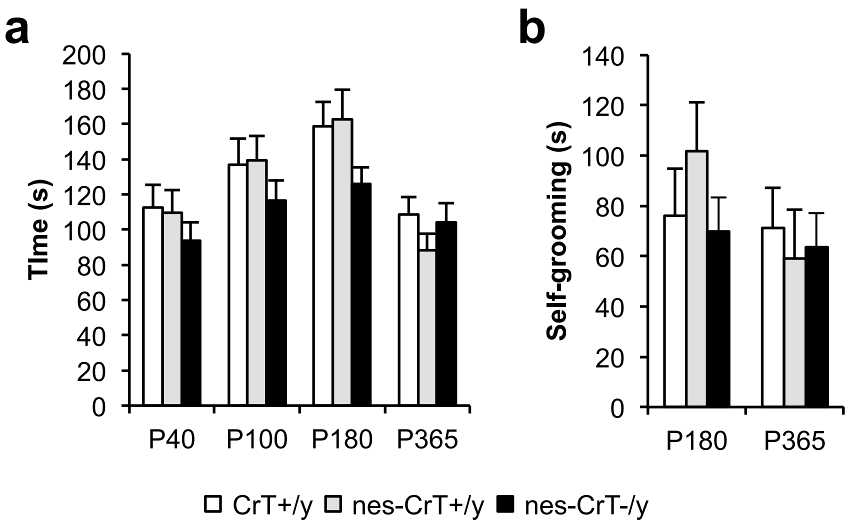

Fig. S8. (a) Longitudinal performance of of $CrT^{+/y}$ mice (n = 13 for P40 and P100, n = 11 for P180 and P365), nes-$CrT^{+/y}$ (n = 9 for P40, P100 and P180, n = 11 for P365) and nes-$CrT^{–/y}$ (n = 8 for P40, P100 and P180, n = 7 for P365) on the accelerating rotarod. The average fall time recorded in the four trials of the test was reported. A Two Way ANOVA on rank transformed data showed no significant effect of genotype ($p = 0.202$, $F(2,105) = 1.625$) and the lack of a statistically significant interaction between genotype and age ($p = 0.671$, $F(6,105) = 0.674$). (b) Histograms display mean time spent self-grooming in $CrT^{+/y}$, nes-$CrT^{+/y}$ and nes-$CrT^{-/y}$ at P180 ($CrT^{+/y}$: n = 11, nes-$CrT^{+/y}$: n = 11, nes-$CrT^{-/y}$: n = 7) and P365 ($CrT^{+/y}$: n = 6, nes-$CrT^{+/y}$: n = 10, nes-$CrT^{-/y}$: n = 7). No difference was detected in grooming behavior at both ages (Two Way ANOVA on rank transformed data, effect of genotype $p = 0.564$ and $F(2,46) = 0.579$, interaction genotype x age $p = 0.131$ and $F(2,46) = 2.125$)

ARRIVE checklist

ITEM	RECOMMENDATION	OUR PAPER
Title	Provide as accurate and concise a description of the content of the article as possible.	A Nervous System-Specific Model of Creatine Transporter Deficiency Recapitulates the Cognitive Endophenotype of the Disease: a Longitudinal Study
Abstract	Provide an accurate summary of the background, research objectives, including details of the species or strain of animal used, key methods, principal findings and conclusions of the study.	193 words
**INTRODUCTION**		
Background	a. Include sufficient scientific background (including relevant references to previous work) to understand the motivation and context for the study, and explain the experimental approach and rationale.<br>b. Explain how and why the animal species and model being used can address the scientific objectives and, where appropriate, the study's relevance to human biology.	See the first two paragraphs of Introduction.
Objectives	Clearly describe the primary and any secondary objectives of the study, or specific hypotheses being tested.	See the last paragraph of Introduction.
**METHODS**		
Ethical Statement	Indicate the nature of the ethical review permissions, relevant licences (e.g. Animal [Scientific Procedures] Act 1986), and national or institutional guidelines for the care and use of animals, that cover the research.	Italian MoH authorization number 259/2016-PR (see Methods)
Study design	For each experiment, give brief details of the study design including: a. The number of experimental and control groups. b. Any steps taken to minimise the effects of subjective bias when allocating animals to treatment (e.g. randomisation procedure) and when assessing results (e.g. if done, describe who was blinded and when). c. The experimental unit (e.g. a single animal, group or cage of animals).	We included details of study design in Methods, Results and Figure Legends.
Experimental procedures	For each experiment and each experimental group, including controls, provide precise details of all procedures carried out.	See Methods and Supplementary material (Behavioral Testing and Biochemical Analysis).
Experimental animals	a. Provide details of the animals used, including species, strain, sex, developmental stage (e.g. mean or median age plus age range) and weight (e.g. mean or median weight plus weight range).<br>b. Provide further relevant information such as the source of animals, international strain nomenclature, genetic modification status (e.g. knock-out or transgenic), genotype, health/immune status, drug or test naïve, previous procedures, etc.	See Methods and Supplementary material. Information about weight is provided in the Result section (Fig. S1).

Housing and Husbandry	Provide details of:<br>a. Housing (type of facility e.g. specific pathogen free [SPF]; type of cage or housing; bedding material; number of cage companions; tank shape and material etc. for fish).<br>b. Husbandry conditions (e.g. breeding programme, light/dark cycle, temperature, quality of water etc for fish, type of food, access to food and water, environmental enrichment).<br>c. Welfare-related assessments and interventions that were carried out prior to, during, or after the experiment.	A detailed description of Housing and Husbandry is provided in Supplementary material (Animals).
Sample size	a. Specify the total number of animals used in each experiment, and the number of animals in each experimental group.<br>b. Explain how the number of animals was arrived at. Provide details of any sample size calculation used.<br>c. Indicate the number of independent replications of each experiment, if relevant.	The sample size for each experimental group is provided in Figure Legends.
Allocating animals to experimental groups	a. Give full details of how animals were allocated to experimental groups, including randomisation or matching if done.<br>b. Describe the order in which the animals in the different experimental groups were treated and assessed.	The allocation of animals to experimental groups is determined by genotype.
Experimental outcomes	Clearly define the primary and secondary experimental outcomes assessed (e.g. cell death, molecular markers, behavioural changes).	See Methods: behavioral tests and Cr levels.
Statistical methods	a. Provide details of the statistical methods used for each analysis.<br>b. Specify the unit of analysis for each dataset (e.g. single animal, group of animals, single neuron).<br>c. Describe any methods used to assess whether the data met the assumptions of the statistical approach.	See Methods (Statistics) and Results.
**RESULTS**		
Baseline data	For each experimental group, report relevant characteristics and health status of animals (e.g. weight, microbiological status, and drug or test naïve) prior to treatment or testing (this information can often be tabulated).	Information about weight is provided in the Result section (Fig. S1).
Numbers analysed	a. Report the number of animals in each group included in each analysis. Report absolute numbers (e.g. 10/20, not 50%2).<br>b. If any animals or data were not included in the analysis, explain why.	The sample size is provided in Figure Legends. Exclusion criteria are described in Supplementary material.
Outcomes and estimation	Report the results for each analysis carried out, with a measure of precision (e.g. standard error or confidence interval).	Results are described using standard error.
Adverse events	a. Give details of all important adverse events in each experimental group.<br>b. Describe any modifications to the experimental protocols made to reduce adverse events.	No adverse events detected.

DISCUSSION

Interpretation/ Scientific implications	a. Interpret the results, taking into account the study objectives and hypotheses, current theory and other relevant studies in the literature.<br>b. Comment on the study limitations including any potential sources of bias, any limitations of the animal model, and the imprecision associated with the results.<br>c. Describe any implications of your experimental methods or findings for the replacement, refinement or reduction (the 3Rs) of the use of animals in research.	See Discussion.
Generalisability/ translation	Comment on whether, and how, the findings of this study are likely to translate to other species or systems, including any relevance to human biology.	New tool to understand the pathogenetic mechanisms underlying neurological deficits induced by Cr deficiency.
Funding	List all funding sources (including grant number) and the role of the funder(s) in the study.	See Acknowledgments
